# A Survey Study of the 3D Facial Landmark Detection Techniques Used as a Screening Tool for Diagnosis of the Obstructive Sleep Apnea Syndrome

**DOI:** 10.3390/arm92040030

**Published:** 2024-08-14

**Authors:** Rastislav Hornák, František Duchoň

**Affiliations:** Institute of Robotics and Cybernetics, Slovak University of Technology, Ilkovičova 3, 84104 Bratislava, Slovakia; frantisek.duchon@stuba.sk

**Keywords:** 3D anatomical landmark detection, obstructive sleep apnea, deep learning, transfer learning, data augmentation, hybrid template and knowledge-based landmark detection

## Abstract

**Highlights:**

As was shown in this paper, OSA screening can be based on facial anatomical landmarks identified in 3D scans. The authors of this paper believe that the amount of quality data necessary for the learning-based methods is too large and therefore see potential in the hybrid template and knowledge-based methods. We plan to use the MeshMonk toolbox in combination with a low-cost Kinect like 3D camera to scan pediatric patients. Automatically identified facial landmarks can not only automate an existing OSA screening protocol but can also help to find new facial features related to the OSA. The ultimate goal is to develop a relatively cheap device, possibly even mobile phone-based, to automatically screen pediatric patients for OSA.

**What are the main findings?**

- OSA screening can be based on facial anatomical landmarks identified in 3D scans.
- Landmark detection based on hybrid templates and knowledge-based methods has significant potential.

**What is the implication of the main finding?**

- Pediatric patients can be screened faster with higher precision.
- New facial features related to the OSA may be discovered.

**Abstract:**

Obstructive Sleep Apnea (OSA) is a common disorder affecting both adults and children. It is characterized by repeated episodes of apnea (stopped breathing) and hypopnea (reduced breathing), which result in intermittent hypoxia. We recognize pediatric and adult OSA, and this paper focuses on pediatric OSA. While adults often suffer from daytime sleepiness, children are more likely to develop behavioral abnormalities. Early diagnosis and treatment are important to prevent negative effects on children’s development. Without the treatment, children may be at increased risk of developing high blood pressure or other heart problems. The gold standard for OSA diagnosis is the polysomnography (sleep study) PSG performed at a sleep center. Not only is it an expensive procedure, but it can also be very stressful, especially for children. Patients have to stay at the sleep center during the night. Therefore, screening tools are very important. Multiple studies have shown that OSA screening tools can be based on facial anatomical landmarks. Anatomical landmarks are landmarks located at specific anatomical locations. For the purpose of the screening tool, a specific list of anatomical locations needs to be identified. We are presenting a survey study of the automatic identification of these landmarks on 3D scans of the patient’s head. We are considering and comparing both knowledge-based and AI-based identification techniques, with a focus on the development of the automatic OSA screening tool.

## 1. Introduction

In this paper, we present a survey study on the automatic 3D facial anatomical landmark detection used in a screening tool for the diagnosis of Obstructive Sleep Apnea (OSA) syndrome. We focus on the diagnosis of pediatric OSA. However, the underlying principles are the same for adult patients as well.

We present various automatic anatomical landmark detection techniques, challenges we face during detection, and approaches to overcome those challenges. The contribution of this paper is that we advocate for the use of hybrid-template and knowledge-based detection techniques. They are more suitable for problems where there is less available training data, typically in medicine. We believe that this approach could also allow faster automation of other than facial anatomical landmarks, which would have a positive impact on the diagnosis of other diseases.

### 1.1. OSA

Obstructive Sleep Apnea (OSA) is a common disorder characterized by repetitive episodes of nocturnal breathing cessations due to upper airway collapse [1]. In general, we distinguish between pediatric and adult patients. According to the authors of [2], pediatric OSA can lead to serious complications, including behavioral abnormalities, neurocognitive impairment, learning disabilities, systemic hypertension and pulmonary hypertension, endocrine and metabolic disorders, maxillofacial dysplasia (adenoid faces), delayed growth and development, and an increase in the risk of cardiovascular events in adulthood. Therefore, early diagnosis and intervention in children with OSA are extremely important. The gold standard for OSA diagnosis is polysomnography (PSG), sometimes called “sleep study”, performed at a sleep center. During the night, oxygen level, brain waves, heart rate, breathing, and also the movement of the legs and eyes are monitored.

### 1.2. Screening, Monitoring, and Early Diagnosis

PSG is an expensive and very stressful procedure, especially for children. Therefore, researchers around the world are looking for alternative and supplemental methods for detecting OSA. There have been different approaches proposed.

#### 1.2.1. Home Monitoring

One way of thinking is to monitor patients’ sleep in the home environment. The use of a camera or cell phone has been suggested. However, according to [3], radar technology seems more promising and does not introduce privacy issues. Doppler radar can be used as a non-contact vital signs monitoring tool able to classify respiratory events such as normal breathing, apnea, and hypopnea. The authors of the paper proposed a heart rate variability (HRV)-based feature extraction technique used in conjunction with traditional ML classifiers like K-nearest neighbors, Random Forest, or Support Vector Machine. In the paper [4], the authors successfully demonstrated the use of a dual-frequency Doppler radar system that integrates sleep posture recognition with cardiopulmonary monitoring, as the momentary sleep posture is vital information for diagnosis and assessment.

Another example of the home monitoring approach would be SNOROSALAB [5]. According to the authors, it is a simple voice-recording device that serves as a screening and diagnostic tool that attempts to detect individuals with suspected snoring and sleep apnea before they enter PSG.

The authors of [6] present a device for treating position and rapid eye movement (REM)-dependent mild and moderate OSA. The device monitors snoring and heart rate. If certain conditions are met (snoring is detected or the heart rate is out of the range of 40–120/min), a vibrational stimulus is sent to the body. The device does not measure the duration or severity of apnea periods but rather reduces OSA periods that occur after snoring periods.

#### 1.2.2. OSA Screening

A different approach is to have a medical professional perform a quick and inexpensive screening test to identify high-risk patients who are best suited for the PSG study.

Often, different questionnaires are used to do so, and a number of them have been developed over time. They are usually based on a specific set of yes-or-no questions used to calculate a score. A big disadvantage of the questionnaires, however, is patients’ subjectivity. Examples of the questionnaires would be:

- STOP—is a concise and easy-to-use screening tool to identify patients with a high risk of OSA [7]. The questionnaire is based on four questions related to Snoring, Tiredness during the daytime, Observed apnea, and high blood Pressure.
- STOP-Bang—is an extended version of the STOP questionnaire, where Bang stands for the following four questions—BMI, Age, Neck circumference, and Gender. According to the authors of the study [7], it has demonstrated a higher sensitivity and negative predictive value, especially for patients with moderate to severe OSA.
- NoSAS—is a questionnaire based on questions related to the following areas: Neck circumference, Obesity, Snoring, Age and Sex. The calculated score ranges from 0 to 17, and the resulting value of 8 or higher means that the patient has a high probability of OSA. According to the authors of [8], the NoSAS score “showed high sensitivity and positive predictive value for OSA with specificity and diagnostic accuracy, which steadily increased with higher scores. Furthermore, a low score showed a high predictive value for the exclusion of moderate/severe OSA”.

Apart from questionnaires, multiple studies have shown that OSA screening tools can be based on facial landmarks and the corresponding shape variables describing facial morphology.

For example, the authors of [9] present a model for predicting OSA using upper airway CT and deep learning. They suggest that their model could even be incorporated into CT procedures to alert clinicians to OSA in patients undergoing head and neck CT.

The authors of [10] studied 653 adult patients undergoing sleep examinations in the Sleep Center of Beijing Anzhen Hospital. The frontal and right profile pictures of each patient were analyzed together with 19 clinical variables (medical history, demographic characteristics, sleep-related symptoms, etc.). The pictures were taken by a single mobile device at a fixed distance and light conditions. The frontal pictures were analyzed by Dlib toolkit to label the facial outline, eyes, nose, and mouth (68 landmarks). According to the study, mandibular features extracted from 2D photos provided efficient and interpretable evidence for the detection of OSA. The OSA group showed increased face height, maxillomandibular height, and mandibular width.

The authors of [11] studied 91 patients with suspected OSA. All underwent a full night PSG at the Multidisciplinary Sleep Disorders Centre of the Antwerp University Hospital and were scanned with the 3dMD head motion system. A set of anatomical landmarks was manually identified, and various distances, angles, and surfaces were calculated. The authors created a model that gave a prediction for OSA severity with an accuracy of 51%. Measurements showed a different course in males and females and in obese and non-obese patients.

The authors of [12] studied a sample population of children in the UK. Initially, 14,541 pregnancies with an estimated date of delivery between April 1991 and December 1992 were selected. Sleep-disordered breathing (SDB) was assessed via parents’ reports of sleep disordered symptoms at the ages of 18, 30, 42, 57, 69, and 81 months for each child. When the children were 15 years old, a total of 1724 children with SDB and 1862 healthy children (1693 males and 1893 females) were selected for further analysis. The study concluded that the combination of a long face, reduced nose prominence and width, and a retrognathic mandible may be diagnostic facial features of SBD. However, the magnitude of the differences between healthy and SDB-diagnosed children is rather small. For example, the increase in face height in SDB children was estimated to be 0.3 mm with a 95% confidence interval (CI) of 0.52 to 0.05. The decrease in mandibular prominence in SDB children was estimated at 0.9°, with a 95% CI of 1.30 to 0.42. To achieve such precision, a strict scanning protocol was implemented. The laser 3D scans were manually annotated, identifying 22 soft tissue facial landmarks, from which 17 face shape variables were calculated. In the end, the measurement precision was estimated to be less than 0.1 mm.

The authors of the paper [13] studied 106 patients. In this group, 50 patients were diagnosed with OSA, and 56 patients were OSA-free. The Artec 3D scanner was used for 3D facial scanning on the same day as the PSG study. On the scans, 12 facial anatomical landmarks were manually identified to evaluate facial morphology. The analyses revealed that there were significant differences between OSA and non-OSA groups. Greater landmark distances were measured in the OSA group, especially in the nasal region. The authors concluded that inter-landmark distances in the craniofacial anatomy might be predictive of OSA.

The authors of the paper [14] performed a study where they combined analyses of craniofacial morphology with other information about the patient, such as anthropometric data, comorbidities, medication, and scores from the BERLIN and NoSAS questionnaires. In total, 13 supervised ML models were trained and compared. They concluded that the combination of 3D geometric morphometrics with ML could serve as an efficient screening tool for OSA.

### 1.3. Cephalometry and Computer-Aided Surgical Simulation (CASS)

Facial landmarks are not used just for OSA screening. There are also other parts of medicine interested in the automation of their identification. Particularly cephalometry and computer-aided surgical simulation (CASS).

Cephalometry is the science of measuring heads in living individuals [15]. Its analytical methods interpret scans called cephalograms to help with diagnostics and treatment planning.

**Figure 1:**
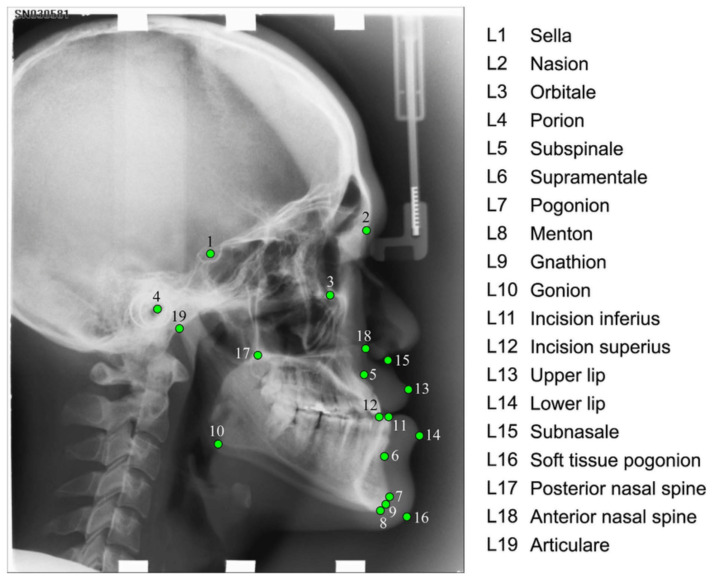
An example of an annotated lateral cephalogram showing all 19 landmark positions used by the authors in their study [17]. The authors aimed to develop and validate an automatic landmark annotation system.

Two-dimensional cephalometry was first introduced in 1931 by Broadbent [16]. It has been using so-called “lateral cephalograms”. The lateral cephalogram is an X-ray image taken from the side of the head with very precise positioning so that various measurements (distances, angles, and proportions) can be performed. A set of landmarks is identified, as shown in the Figure 1 below.

Lateral cephalograms are still widely used; however, 3D alternatives are available now. To get a 3D picture computerized tomography (CT) data may be converted into a 3D image, or cone beam computed tomography (CBCT) can provide a 3D image directly. When either a 2D lateral cephalogram or a 3D image is taken, the landmarks have to be identified and annotated. This is often performed manually. It requires an experienced person and is slow, labor-intensive, and tedious work. Therefore, any automation is of great help. Recently, multiple studies have been conducted to evaluate the clinical feasibility of deep learning based CBCT image segmentation in computer-aided surgical simulation (CASS). The authors of [18] validated SkullEngine and concluded that it is ready for daily practice; however, the accuracy needs to be improved. The overall mean error was estimated to be 2.3–2.4 mm.

Cephalometry, in general, focuses on the patient’s head. However, landmark detection can also be useful for other parts of the human body. For example, the authors of the study [19] focused on the anatomy of the femur.

Complex surgeries, such as the treatment of patients with complex craniomaxillofacial (CMF) deformities, can be planned using CASS [20]. In general, the use of CASS has greatly improved the efficiency and accuracy of orthognathic surgeries. An image of the skeleton structure acquired either from CT or CBCT is to be segmented, i.e., each anatomical structure has to be identified and labeled. This is performed by 3D cephalometry [21,22].

## 2. Anatomical Landmarks

According to [23], anatomical landmarks are defined as biologically meaningful loci that can be unambiguously defined and repeatedly located with a high degree of accuracy and precision. Landmark data are useful because there are now a multitude of methods for the statistical analysis of forms using landmark data, as well as because more traditional measures like linear distances and angles can be calculated from the landmark data.

### 2.1. Detection Precision

The analytical results of any data can only be as good as the data itself. Therefore, attention has to be paid to the measurement errors too. In the papers [24,25], the following evaluation metrics are used:

1. Mean radial error—Euclidian distance between the position of the estimated landmark and the manually annotated landmark.
2. Success detection rate—if a landmark has been evaluated with a radial error less than a certain threshold epsilon, then it is considered a successful detection. The success detection rate is then the number of successful detections divided by the number of all detections.
3. Standard deviation—standard deviation of Euclidian errors of the estimated landmarks.

According to [26], a 2 mm precision range is the acceptable precision range in clinical practice. That means that the distance between the automatically identified landmark and the manually identified landmark should be equal to or less than 2 mm.

### 2.2. Detection Techniques

There are a variety of landmark detection techniques that have been developed. These can be categorized based on different criteria. For example, the authors of the studies [19,26] have divided them into the following two groups:

1. Knowledge-based techniques: these techniques use pre-existing information about the system. Here belong Active Shape Models (ASMs) and Active Appearance Models (AAMs), edge and pattern detection techniques, as well as genetic programming.
2. AI-based techniques include machine learning and deep learning.

**Figure 2:**
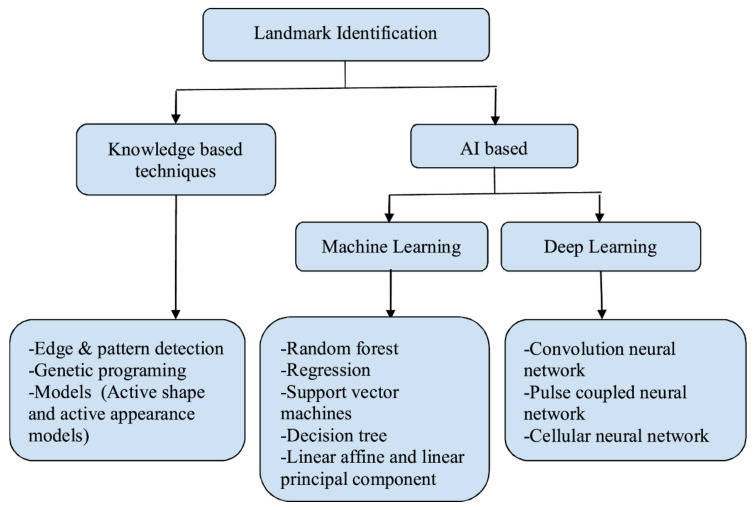
The categorization of landmark identification techniques is presented in the study [26]. The authors have divided the techniques into two major categories: knowledge-based techniques and AI-based techniques.

Each group was further divided into different subgroups which is nicely illustrated in the Figure 2.

According to the authors of the paper [27], methods of landmark identification and annotation can be classified into three categories:

1. Knowledge based methods use feature extraction, edge detection, etc. Geometry and overall anatomical characteristics are used.
2. Template-based methods use statistical models as reference templates that are fitted or matched with the target anatomical structure.
3. Learning-based methods use machine learning, neural networks, and deep learning algorithms.

The authors of the paper [28], focusing on human head modeling, have divided landmark detection methods into these two categories.

1. Indirect methods

Here, 3D data are rendered and projected into the 2D world to get a 2D image. Then, in this image, 2D facial landmarks are detected. Then, to get into the 3D world, the corresponding points from the original 3D data are estimated. The advantage of this approach is that already mature deep learning-based 2D landmark detection methods are used. Therefore, good results can be achieved.

2. Direct methods

Three dimensional data in the form of a facial mesh or point cloud is taken as input, and identified facial landmarks are returned directly. There is no middle step, as in the indirect methods.

## 3. Challenges

There are multiple reasons why automating the identification of anatomical landmarks is a challenging task. To name a few:

- The clinical practice defines which anatomical landmarks need to be identified. Only specific locations-anatomical landmarks-are to be identified, not arbitrarily chosen ones. For example, the authors of [19] proposed an automatic algorithm for detecting a specific set of anatomical landmarks on the human femur, which served as input for the calculation of various metrics (distance, angle, and volume) aimed at detecting supracondylar fractures.
- In computer vision, we mostly focus on the face as the most interesting part of the human body. However, anatomical landmarks can be located anywhere on or in the body. These other areas are not as extensively studied, so we usually do not have much training data. Moreover, it is often unrealistic to obtain thousands of images or scans of a particular body part. Even when scans are available, they must be annotated manually by a medical professional, which is expensive and impractical. Therefore, the authors of [19] developed a knowledge-based approach for automatically identifying femur anatomical landmarks that does not require training data.
- According to the authors of [11], it is difficult to locate some landmarks without tactile confirmation, i.e., without palpation of the underlying bony structure.

In our case, we focus on the face. This brings some specific challenges, like:

- Variability in facial expressions mimics.
- Occlusions by hair, glasses, or other objects.
- Face diversity due to age, gender, ethnicity, and injuries.
- Some diseases have an impact on facial structure, typically Down syndrome.

## 4. Approaches

There have been multiple approaches developed to overcome the challenges mentioned in the previous chapter.

### 4.1. Strict Data Acquisition Protocol

Some variations in data can be minimized by using a strict data acquisition protocol, such as controlling for occlusions or facial expressions.

### 4.2. Transfer Learning and Data Augmentation

Deep learning (DL) has outperformed other computer vision techniques in many applications. Its hunger for data, however, poses a big problem for medical applications where training data are not easily available. The solution to this challenge used by the authors of the paper [29] is transfer learning (TL) and data augmentation (DA). Their objective was to develop and evaluate algorithms for the automated extraction of orofacial landmarks that characterize airway morphology. They defined 27 frontal + and 13 lateral landmarks and were able to collect *n* = 317 pairs of pre-surgery photos from patients undergoing general anesthesia (140 females and 177 males). The landmarks were independently annotated by two anesthesiologists to get a reference for the supervised learning. The authors then trained two deep convolution neural networks (DCNNs) [29] with this data, one for the frontal view and one for the later view. Their DCNN’s had worked with 2D inputs; however, the authors concluded that in the future they will be focusing on the estimation of 3D landmarks.

Transfer learning is an approach where a DL network is first trained with other data than the final training set, or even a publicly available network is used in this step. Then this knowledge is reused when training with the final training set is performed.

To extend the training dataset, data augmentation was performed. It was implemented as a combination of various transformations of the input images-zoom, rotation, shear, flip, color shift, etc.

### 4.3. Hybrid Template and Knowledge-Based Approach

Another approach to dealing with the described challenges is the template- and knowledge-based approach.

The authors of the paper [27] proposed a hybrid template- and knowledge based method to locate landmarks in 3D surface models of skulls. Over thirty 3D scans of male Caucasian skulls were used to validate the approach. On each scan, 58 craniometric landmarks were annotated in a two-step process. 

First, a deformable template was used to estimate the initial landmark positions. This procedure followed a template-based strategy where a reference 3D template was aligned to the target mesh model of the skull. The publicly available MeshMonk registration toolbox, which performs non-rigid registration, was utilized for this purpose [30]. Then the refinement step followed. The previously estimated landmark positions were further improved using prior anatomical knowledge in the form of geometric cues. Refinement was bounded by a region of interest established for each landmark. The following set of geometric cues (heuristics) was employed:

- Symmetry
- Contour information
- Local curvature
- Ridges
- Instrumental

A statistical analysis of the variability of manual and automatic annotations was performed with the following results:

Inter-observer and intra-observer manual annotation errors were estimated to have a mean of 1.62 ± 1.2 mm.

The automatic annotation error was estimated to have a mean of 2.25 ± 1.6 mm, and it was observed that the results were profoundly influenced by the quality and accuracy of the underlying 3D model.

In 2021, authors published a study [31] that focused on automatic craniomaxillofacial 3D dense phenotyping. The study elaborates on the reliability and accuracy of the automatic phenotyping approach employing the MeshMonk toolbox. Phenotyping was performed on 30 unaltered and 20 altered, i.e., operated, human mandibles. The MeshMonk toolbox performed non-rigid registration of a template mesh, which consisted of 17,415 landmarks. The authors called these landmarks “quasi landmarks” to distinguish them from the anatomical landmarks. Out of these 17,415 quasi-landmarks, 26 corresponded to actual anatomical landmarks. Anatomical landmarks were also annotated manually by seven observers. Repeated measurements confirmed excellent automatic repeated-measures reliability. The annotation error between manual and automatic measurements was estimated to have a mean of 1.4 mm for the unaltered mandibles and 1.76 mm for the operated mandibles.

In 2024, authors published a paper [32] in which the MeshMonk toolbox was used to phenotype craniofacial bone on a dataset of 31 sculls. A cone-beam computed tomography (CBCT) scan was taken from the skulls. A single surface craniofacial bone mask consisting of 9999 quasi-navigations was used in MeshMonk as a template. Out of all the quasi-landmarks, 20 corresponded to the actual anatomical landmarks. Three observers identified the 20 landmarks manually. The mean distance between automatically and manually identified landmarks was 1.5 mm, ranging from 0.1 mm to 7.2 mm. 

#### 4.3.1. MeshMonk Toolbox

MeshMonk was introduced in the paper [33] in 2019 as an open-source toolbox for 3D phenotyping to facilitate the integration of genomic and phenomic data. The MeshMonk has been implemented in C++ with a focus on speed. It performs dense surface registration. Initially, a surface template is aligned with the target surface using the iterative closest point algorithm, allowing only translation, rotation, and isotropic scaling of the template. A non-rigid transformation is performed in the next step so that, in the end, the template shape matches the target surface. The overall performance can be optimized by adjusting different parameters.

To analyze the performance of the toolbox, the authors used stereo photogrammetry to capture approximately 6000 faces in the format of 3D point clouds. From this large dataset, a random sample of 41 faces was chosen. The sample images exhibited diversity in terms of gender, age, height, and the 3D camera system employed. Manual annotation of 19 validation landmarks was performed by two independent observers three times each, with at least 24 h between the sessions. This means that each validation landmark was marked six times. The average location of each validation landmark for each face was calculated next. Each face was registered on the template, and then the average locations of the validation landmarks were transferred onto the template. This makes 41 × 2 observers × 3 sessions = 246 manual landmark positions on the template. Out of 41 faces, one was selected as a test face. For the rest of the 40 faces, the manual land mark positions transferred to the template were averaged, and these averages were transferred to the single excluded face. This way, the validation landmarks were automatically placed on the test face without any bias. This process was repeated for each of the 41 faces, so each face was excluded once while the others were processed. 

The authors reported that the mean distance between automatically and manually annotated landmarks was from 0.70 to 1.68 mm.

#### 4.3.2. TH-OCR

In 2023, the authors of the paper [34] presented a comparison study evaluating the performance of the MeshMonk toolbox and their own TH-OCR algorithm. They collected 3D facial scans of 20 males. A set of 32 facial anatomical landmarks was located on each facial scan using both the MeshMonk toolbox and the TH-OCR algorithm. The same 32 facial anatomical landmarks were also located manually by experts, and the Euclidian distance between the manually and automatically identified anatomical landmarks was calculated.

The mean Euclidian distance in the MeshMonk group was estimated to be 2.16 ± 1.97 mm.

The mean Euclidian distance in the TH-OCR group was estimated to be 2.34 ± 1.76 mm.

### 4.4. Thresholded Surface Normals

The authors of the paper [35] proposed a 3D facial landmarking algorithm based on thresholded surface normals. They focused on the identification of key nasal landmarks. The algorithm is inspired by the successful use of surface normals for feature extraction. Surface normals provide an effective representation of the surface geometry. Among the benefits of using surface normals are high consistency and accuracy under different facial expressions. Their algorithm is training-free and computationally very fast.

## 5. Discussion

### 5.1. Comparison with Previous Research

In our work, we aim to use 3D facial scans of the patients performed by a cheap 3D Kinect-like camera. Previous studies mostly used 2D pictures or expensive CT scans and 3D scanners. In our work, we also want to avoid training the ML or DL models, as this requires a huge amount of training data. Since facial morphology varies a lot across age, sex, and ethnicity, it would be difficult to have all these variations included in the training set. 

### 5.2. Limitations

Despite the promising findings, this study has several limitations. First, we have not tested any specific hybrid template or knowledge-based algorithm (neither the MeshMonk toolbox nor TH-OCR) on the actual facial scans created by a Kinect like 3D camera. Second, the facial scans considered in this paper do not capture intraoral morphology, which can provide important signs and symptoms associated with OSA.

### 5.3. Future Research

The MeshMonk toolbox, in conjunction with a low-cost 3D camera like Kinect, could be utilized to scan pediatric patients. Automatically identified facial landmarks can not only streamline existing OSA screening protocols but also aid in discovering new facial features associated with OSA. The ultimate goal is to develop a relatively cheap device, possibly even mobile phone-based, to automatically screen pediatric patients for OSA.

### 5.4. Conclusions

The authors of this paper have demonstrated that screening and early diagnosis of OSA are crucial yet challenging tasks. Facial anatomical landmarks can be used for screening, with automation of their identification being a vital component. The amount of quality data required for the learning-based methods is substantial; hence, the authors see potential in hybrid templates and knowledge-based methods. 

## Data Availability

No new data were created or analyzed in this study. Data sharing is not applicable to this article.
